# Embryo production in the sponge-dwelling snapping shrimp *Synalpheus apioceros* (Decapoda, Alpheidae) from Bocas del Toro, Panama

**DOI:** 10.3897/zookeys.457.6403

**Published:** 2014-11-25

**Authors:** Adriana P. Rebolledo, Ingo S. Wehrtmann, Darryl L. Felder, Fernando L. Mantelatto

**Affiliations:** 1 Unidad de Investigación Pesquera y Acuicultura (UNIP) of the Centro de Investigación en Ciencias del Mar y Limnología (CIMAR), Universidad de Costa Rica, 11501-2060 San José, Costa Rica; 2 Department of Biology, Laboratory for Crustacean Research, University of Louisiana-Lafayette, Lafayette, LA 70504-2451, USA; 3 Laboratory of Bioecology and Crustacean Systematics, Department of Biology, FFCLRP, University of São Paulo, Postgraduate Program on Comparative Biology, Av. Bandeirantes 3900, 14040-901, Ribeirão Preto, São Paulo, Brazil

**Keywords:** Central America, embryo volume, fecundity, incubation period, reproductive output, water uptake

## Abstract

Caridean shrimps of the genus *Synalpheus* are abundant and widely distributed in tropical and subtropical regions, but knowledge of their reproductive biology remains scarce. We report reproductive traits of *Synalpheus apioceros* from Bocas del Toro, Panama, based on collections in August 2011. The 46 ovigerous females that were analyzed ranged in size from 3.8 to 7.4 mm in carapace length. Fecundity varied between 8 and 310 embryos and increased with female size. Females invested 18.6 ± 10.3% of their body weight in Embryo production. Embryo volume increased considerably (77.2%) during embryogenesis, likely representing water uptake near the end of incubation period. Compared to *Synalpheus* species with abbreviated or direct development, *Synalpheus apioceros* produced substantially smaller embryos; however, *Synalpheus apioceros* seems to have a prolonged larval phase with at least five zoeal stages, which may explain the combination of relatively small and numerous embryos. We did not find nonviable, minute, chalky embryos, previously reported for *Synalpheus apioceros* specimens obtained from the northwestern Gulf of Mexico, which supports the hypothesis that the production of this type of embryos may be a physiological response of this warm-water species to the temperature decrease near to its latitudinal range limit.

## Abstract (es)

Aunque los camarones carídeos del genero *Synalpheus* son muy abundantes y se encuentran ampliamente distribuidos en regiones tropicales y subtropicales, el conocimiento sobre su biología reproductiva es escaso. En este estudio reportamos algunas características reproductivas de especímenes de *Synalpheus apioceros* de Bocas del Toro, Panamá, colectados en Agosto del 2011. El largo del caparazón de las 46 hembras ovígeras analizadas se encuentra en un rango de 3.8 y 7.4 mm. La fecundidad varió entre 8 y 310 huevos, aumentando con el tamaño de la hembra. Las hembras invirtieron 18.6 ± 10.3% de su peso corporal en la producción de huevos. El tamaño de los huevos aumentó considerablemente (77.2%) durante la embriogénesis, probablemente por la absorción de agua al final del periodo de incubación. Comparado con especies de *Synalpheus* que presentan desarrollo abreviado o directo, *Synalpheus apioceros* produce huevos considerablemente más pequeños; sin embargo *Synalpheus apioceros* parece tener una fase larval prolongada, con al menos cinco estadios larvales, lo que podría explicar que los huevos sean relativamente pequeños y numerosos. No encontramos los huevos anómalos, no viables, que previamente se habían reportado para especímenes obtenidos en el Golfo de México, lo cual apoya la hipótesis de que la producción de este tipo de huevos puede ser una respuesta fisiológica a la disminución de temperatura cerca del límite latitudinal de esta especie habitante de aguas cálidas.

## Introduction

Reproductive traits of crustacean species offer relevant information about their life history strategies (Sastry 1983, Ramirez-Llodra 2002). Fecundity, defined as the number of offspring produced by a female in a determined time period, is directly related to energy allocation and is essential in estimating reproductive potential of a population (Ramirez-Llodra 2002, Zare et al. 2011). Embryo size is an indicator of the energy allocation, duration of embryogenesis and type of larval development (Dardeau 1984, Ramirez-Llodra 2002). The reproductive output quantifies the energetic investment of a species in embryo production (Hines 1991, Anger and Moreira 1998).

Caridean shrimps of the genus *Synalpheus* are distributed worldwide with estimates of about 150 valid species (Dardeau 1984, Ríos and Duffy 2007, Macdonald et al. 2009, Hermoso-Salazar and Solis-Weiss 2010, Hultgren et al. 2010, 2011, De Grave and Fransen 2011, Anker et al. 2012). Whether free-living or (more typically) living as facultative or obligate symbionts, these pistol or snapping shrimps commonly inhabit sponge cavities, coral reefs, rocks, grass beds, or tide pools, and may live associated with hard or soft corals, ascidians, bryozoans, and crinoids (Corey and Reid 1991, Ríos and Duffy 2007, Macdonald et al. 2009, Anker et al. 2012).

Most studies on this genus have focused on geographical distribution (Hermoso-Salazar and Hendrickx 2005a, Macdonald et al. 2009, Bacci et al. 2010, Hultgren et al. 2011), taxonomy and species revision (Duffy 1996a, Ríos and Duffy 1999, Hermoso-Salazar et al. 2005, Hermoso-Salazar and Hendrickx 2005a,b, Macdonald and Duffy 2006, Anker and Toth 2008, Anker et al. 2012), phylogenetics (Morrison et al. 2004, Hultgren and Duffy 2011), or behavior and eusociality (Duffy 1996b,^1998^, Duffy and Macdonald 1999, Duffy et al. 2000, 2002). Despite the large amount of information accumulated about these interesting snapping shrimps, little is known about their reproductive ecology. Corey and Reid (1991) provided some data on the fecundity of six species of *Synalpheus*, and Hernáez et al. (2010) described reproductive features and the effect of bopyrid parasitism on embryo production in *Synalpheus yano* Rios & Duffy. Dobkin (1969) studied the larval development of *Synalpheus apioceros* Coutière and concluded that this species has a prolonged larval phase. The only published study regarding embryo production of *Synalpheus apioceros* mentioned the presence of anomalous small embryos and described the pattern of seasonality of ovigerous females from the northwestern Gulf of Mexico but did not address fecundity (Felder 1982).

*Synalpheus apioceros* is widely distributed in the western Atlantic (Gulf of Mexico; Florida; Bahamas), throughout the Caribbean Sea (e.g., Panama, Puerto Rico, Mexico, Venezuela etc.), Suriname, and Brazil (Amapá to Santa Catarina). Assuming that we are dealing with a single species, it can be found in association with different hosts (see Anker et al. 2012 for revision). The species thus represents an excellent candidate in to study reproductive adaptations of those *Synalpheus* spp. that typically live in heterosexual pairs but with a variety of hosts. The present study addresses reproduction in *Synalpheus apioceros* by describing fecundity, reproductive output, volume and water content of the embryos at different embryonic stages for a tropical Caribbean population.

## Methods

Ovigerous females of *Synalpheus apioceros* were collected by hand (August 2011) from an area near the Smithsonian Tropical Research Institute (STRI) marine station (09°20'N, 82°14'W), at Bocas del Toro, on the Caribbean coast of Panama. Shrimps were found in the red-orange sponge *Lissodendoryx colombiensis* Zea & van Soest, growing on jetty pilings and mangrove roots. In the laboratory, ovigerous females were extracted from the sponge canals and stored individually to avoid mixing and loss of the embryos and then preserved in ethanol (70%). Voucher specimens were deposited in the Crustacean Collection of the Museo de Zoología - Universidad de Costa Rica (MZUCR) under catalog number MZUCR 3128-01.

Carapace length (CL) of ovigerous females was measured (± 0.1 mm) under a stereomicroscope with an ocular micrometer, from the tip of the rostrum to the posterior margin of the carapace. The entire embryo mass from each female was detached from the pleopods and embryos were classified into three stages (I–III) according to the shape and development of the abdomen and eyes (Wehrtmann 1990): Stage I: almost round embryo, uniform yolk, no visible eye pigments; Stage II: ovoid embryo, eye pigments barely visible; Stage III: ovoid embryo, eye fully developed, abdomen free.

Ten embryos of each female were randomly selected to measure the length (*a*) and width (*b*) under a stereomicroscope equipped with an ocular micrometer; these data were used to determined the embryo volume (EV) with the formula EV = 1/6×*a*×*b*^2^×π (Turner and Laurence 1979). The wet weight (WW) of the embryo mass was determined with an analytical balance and the embryos then oven-dried at 60 °C for 24 hours to obtain embryo mass dry weight (EDW). Embryo water content was calculated by subtracting EDW from WW.

Due to possible embryo loss during the incubation period (Terossi et al. 2010), only females carrying recently-produced embryos (Stage I) were used to estimate fecundity and reproductive output (RO). Fecundity was considered as the total number of embryos present on each individual. The dry weight of the females (FDW) carrying Stage I embryos was obtained to calculated the RO applying de formula proposed by Clarke et al. (1991): RO = EDW_(Stage I)_/FDW.

Data were analyzed with the statistical software SPSS v.20.0. The assumption of normality on the size distribution of the individuals was tested using the Kolmogorov-Smirnov test. Linear regressions and Pearson’s correlation analyses were applied to determine the relation between CL and fecundity, and CL and RO. One-way Analyses of Variance (ANOVA) were used to compare embryo volume and water content among the three stages of development.

## Results

**Figure 1.**
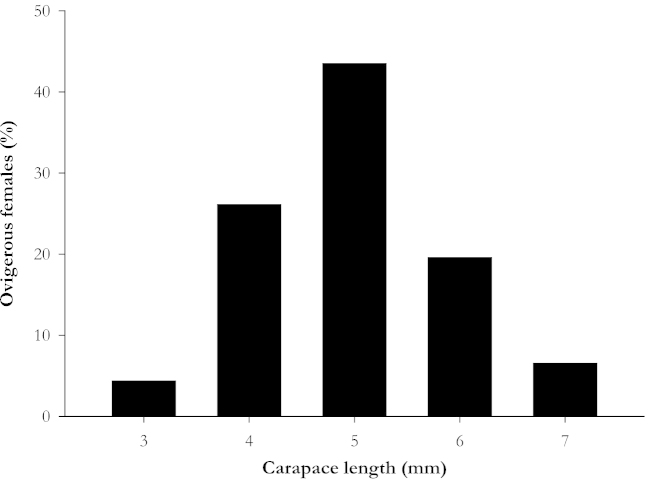
Frequency distribution of carapace length of ovigerous females of *Synalpheus apioceros* (N = 46), Bocas del Toro, Panama.

A total of 46 ovigerous females were analyzed; the majority of them (21 total or 45.7%) carried embryos at Stage I, 10 (21.7%) in Stage II and 15 females (32.6%) in Stage III. The size frequency distribution was normal (Kolmogorov-Smirnov test, *KS* = 0.11, *p* > 0.05). The mean CL of the individuals was 5.4 ± 0.8 mm, ranging from 3.8 to 7.4 mm, and 43.5% of the ovigerous females were in the intermediate size class of 5.0–5.9 mm (Fig. 1).

**Figure 2.**
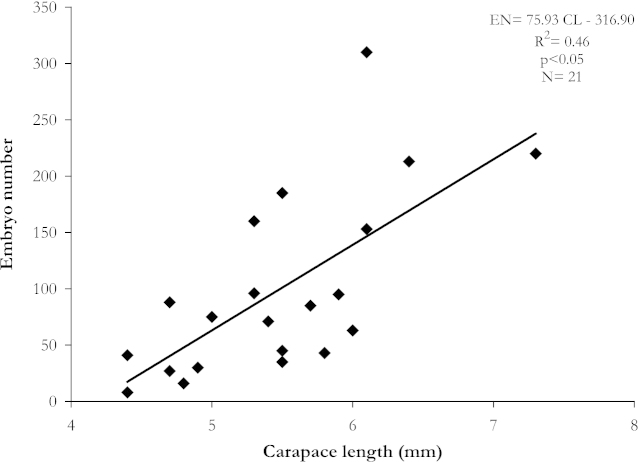
Relation between carapace length and fecundity of *Synalpheus apioceros* females carrying stage I embryos, Bocas del Toro, Panama.

Fecundity in Stage I ranged from 8 to 310, and increased with female size (Pearson’s correlation, *r* = 0.68, *p* < 0.05) (Fig. 2). Females within the same size-class presented different number of embryos (Fig. 2 and Table 1). Energy invested in embryo production by the females was not related to female size (Pearson’s correlation, *r* = 0.26, *p* > 0.05). The embryo mass comprised on average 18.6 ± 10.3% (4.8–40.1%) of the dry body weight of the females.

**Table 1.** Fecundity by size class in females carrying recently-produced embryos (Stage I) for *Synalpheus apioceros*, Bocas del Toro, Panama.

Size class (mm)	Mean embryos number
4.0–4.9 (N = 6)	34.8 ± 28.5
5.0–5.9 (N = 10)	88.6 ± 49.6
6.0–6.9 (N = 4)	184.8 ± 103.8
7.0–7.9 (N = 1)	220

Embryos were slightly oval with mean diameters ranging from 0.63 ± 0.04 mm (Stage I) to 0.77 ± 0.06 mm (Stage III). Embryo volume differed significantly (ANOVA, *F* = 369.25, *p* < 0.05) between the stages of development, with an overall volume increase during the incubation period of 77.2% (Table 2). During the embryogenesis, the water content increased substantially (ANOVA, *F* = 82.60, *p* < 0.05) from Stage I (59.0 ± 5.9%) to Stage III (82.9 ± 3.6%). Dry mass remained almost constant throughout the incubation period (ANOVA, *F* = 0.10, *p* > 0.05) (Table 2).

**Table 2.** Embryo volume, weight, and water content of different embryonic development stages of *Synalpheus apioceros*, Bocas del Toro, Panama.

Embryo features	Stage I (N = 20)	Stage II (N = 8)	Stage III (N = 9)
Embryo volume (mm^3^)	0.101 ± 0.015	0.136 ± 0.018	0.179 ± 0.034
Wet weight (µg)	74.0 ± 12.5	128.2 ± 10.5	173.9 ± 19.8
Dry weight (µg)	30.1 ± 5.3	29.8 ± 1.5	29.3 ± 5.2
Water content (µg)	43.9 ± 10.0	98.4 ± 9.4	144.6 ± 21.0
% Water content	59.0 ± 5.9	76.6 ± 2.0	82.9 ± 3.6

## Discussion

The size of ovigerous females of *Synalpheus apioceros* from Bocas del Toro is within the range for congeneric species (Table 3). Fecundity in *Synalpheus apioceros* increased with female size, which is in agreement with similar observations from other caridean shrimp (Corey and Reid 1991, Anger and Moreira 1998, Lara and Wehrtmann 2009). Female size is postulated to determine the number of embryos produced by the individual, as larger females have more space available for the attachment of embryos on their pleopods (Lara and Wehrtmann 2009).

**Table 3.** Mean carapace length, embryo number, and embryo volume for ten species of sponge-dwelling *Synalpheus*.

Species	N	Carapace length (mm)	Embryo number	Embryo volume (mm^3^)	Reference
*Synalpheus agelas*	5	5.0 (4.2–5.6)	42.4 (16–65)	0.23	Corey and Reid (1991)
*Synalpheus brooksi*	10	3.9 (3.4–4.5)	5.8 (3-11)	0.50	
*Synalpheus fritzmuelleri*	13	4.9 (3.8–6.5)	173.4 (39–484)	0.09	
*Synalpheus herricki*	4	4.5 (3.5–5.12)	45.8 (11–81)	0.22	
*Synalpheus longicarpus*	21	6.9 (5.5–8.0)	195.4 (27–349)	0.17	
*Synalpheus pectiniger*	31	4.2 (3.5–4.6)	9.9 (4–17)	0.75	
*Synalpheus chacei*	2	3.7	16	0.15	Hernáez et al. (2010)
*Synalpheus idios*	4	3.9 ± 0.2 (3.7–4.2)	12.0 ± 4.3 (9–18)	1.04 ± 0.23 (0.70–1.20)	
*Synalpheus yano*	84	5.6 ± 1.2 (3.7–9.6)	98.0 ± 64.6 (6–246)	0.15 ± 0.08 (0.06–0.51)	
*Synalpheus apioceros*	46	5.4 ± 0.8 (3.8–7.4)	97.8 ± 80.1 (8–310) N = 21 (Stage I)	0.13 ± 0.04 (0.07–0.24) N = 37	Present study

Fecundity variation within the same size class is a well-known phenomenon in decapods, including alpheid shrimps (Lardies and Wehrtmann 1997, 2001, Pavanelli et al. 2008). Such variability as observed in *Synalpheus apioceros* could be due to multiple spawnings during a single reproductive season (Miranda et al. 2006, Mantelatto et al. 2007). Also, the presence of primiparous females, which tend to produce fewer embryos than multiparous ones (Somerton and Meyers 1983, Mantelatto et al. 2007) could explain the variability of embryo number within the same size class.

The energy invested in embryo production, estimated by reproductive output (RO), was not related to their size in *Synalpheus apioceros*. Average RO in alpheid shrimp is highly variable, ranging from 7 to 35% (Lardies and Wehrtmann 1997, 2001, Pavanelli et al. 2008, 2010, Hernáez et al. 2010). Although the RO of *Synalpheus apioceros* is lower (18.6%) than that reported for another tropical sponge-dweller, *Synalpheus yano* (35%: Hernáez et al. 2010), it is higher than that observed in other subtropical alpheid species (Pavanelli et al. 2008, 2010). The elevated RO values in sponge-dwelling alpheids may represent an adaptation to their life style: the relatively protected habitat may favor energy investment in embryo production, which also favors enhanced larval production and thus probability of recolonization in niches restricted to certain hosts. This interpretation is supported by studies on pinnotherid crabs living in different hosts, which showed extremely high RO values (*Pinnotheres ostreum* Say: 66%; *Fabia subquadrata* Dana: 97%; Hines 1992). Thus, it might be postulated that decapod species living associated with other organisms generally have a higher RO than free-living species, a pattern warranting additional studies.

The embryo volume in *Synalpheus apioceros* is within the range reported for other alpheid shrimp (Corey and Reid 1991, Lardies and Wehrtmann 1997, Pavanelli et al. 2008, 2010, Hernáez et al. 2010). Several other species of *Synalpheus* produce substantially larger embryos than does *Synalpheus apioceros* (see Corey and Reid 1991), but these species have an abbreviated or direct development. In contrast, *Synalpheus apioceros* seems to have a prolonged larval phase with at least five zoeal stages (Dobkin 1969), which may explain the presence of relatively small and numerous embryos.

Felder (1982) studied reproductive features of *Synalpheus apioceros* in the northwestern Gulf of Mexico (26°49.0'N, 97°19.3'W), close to the northern limit of the geographical distribution range of the species. He reported the presence of nonviable, minute, chalky embryos, and suggested that the production of this type of embryos may be a physiological response of this warm-water species to the temperature decrease near to its latitudinal range limit. This hypothesis is perhaps supported by the present data, because we did not detect in any of the females examined herein this type of minute embryos. We propose that laboratory experiments with *Synalpheus apioceros* be conducted to determine if lowered maintenance temperatures can provoke the production of these nonviable, minute embryos and if higher temperatures can alternatively eliminate their occurrence. Should ongoing elevation of temperatures in northern extremes of range for these tropical decapods reduce production of non-viable embryos, and thus enhance effective fecundity, this could reveal an underappreciated dynamic of coastal ocean warming.

Embryo volume of *Synalpheus apioceros* increased considerably (77.2%) during the incubation period. This is a common phenomenon in decapod species and is probably related to water uptake over the course of embryogenesis (Lardies and Wehrtmann 1997, Petersen and Anger 1997, Wehrtmann and Lardies 1999, Lara and Wehrtmann 2009), as also observed incrementally in *Synalpheus apioceros* (Table 2). At the end of the incubation period, the embryo starts to swell due to osmotic changes (Figueiredo et al. 2008), while the embryo membrane shows a decrease in thickness with a concomitant increase in elasticity, thus favoring the hatching process of the embryo (Davis 1981).

While we here provide novel information on reproduction in the sponge-dwelling alpheid shrimp *Synalpheus apioceros*, it is based on a limited sampling period and a single locality. We thus regard our work to date as a starting point from which we and others might build comparative studies. Conspecific populations can be readily sampled across latitudes and temperature regimes, as well as over varied seasons, applying the methods we have used and enabling comparative analyses. Such work can both reveal life history strategies that have evolved in these host-dependent shrimp species and shed light on what ranges of reproductive variability might be expected due to environmental interactions in this era of global coastal ocean change.
